# Parathyroid hormone enhances appetite and fails to reduce adiposity in ob/ob mice

**DOI:** 10.1007/s00424-026-03183-y

**Published:** 2026-06-03

**Authors:** Abul Fajol, Hirotaka Komaba, Chigusa Ishioka, Yosuke Nakagawa, Naoto Hamano, Takehiko Wada, Masafumi Fukagawa

**Affiliations:** 1 Division of Nephrology, Endocrinology and Metabolism, Tokai University School of Medicine, 143 Shimokasuya, Isehara, 259-1193 Japan; 2 The Institute of Medical Sciences, Tokai University, Isehara, Japan

**Keywords:** Adipose tissue browning, Diabetes, Ghrelin, Obesity, Parathyroid hormone

## Abstract

Obesity is a major driver of type 2 diabetes, underscoring the urgent need for therapies that target both energy expenditure and glucose homeostasis. Parathyroid hormone (PTH) has been implicated in promoting adipose tissue browning and thermogenesis, but its metabolic impact in the context of obesity remains uncertain. We aimed to examine the effects of PTH in ob/ob mice, a leptin-deficient model of obesity and diabetes. A single dose or daily injections of human PTH(1–34) were administered to ob/ob mice and their wild-type littermates. Metabolic outcomes, adipose tissue thermogenesis, and appetite-regulating pathways were assessed. A pair-feeding study was also performed to isolate the effect of PTH from food intake. A single acute dose of PTH upregulated thermogenic gene expression in adipose tissue in a dose-dependent manner. However, repeated daily PTH administration failed to sustain adipose browning or improve insulin resistance and hyperglycemia; instead, it exacerbated weight gain, primarily due to increased food intake. Mechanistically, PTH enhanced gastric ghrelin production and upregulated hypothalamic *AgRP* expression, suggesting a central orexigenic effect. In pair-feeding experiments, PTH-treated mice showed no significant improvements in adiposity or metabolic parameters, even when caloric intake was matched. Although PTH transiently promotes thermogenesis in adipose tissue in ob/ob mice, sustained exposure exacerbates hyperphagia without meaningful metabolic benefit. These findings highlight the challenges of targeting PTH signaling for treating obesity-related diabetes.

**Supplementary Information:**

The online version contains supplementary material available at 10.1007/s00424-026-03183-y.

## Introduction

Obesity is a growing epidemic that contributes to numerous chronic diseases, including type 2 diabetes, cardiovascular disease, and chronic kidney disease [1]. The primary cause of obesity is a positive energy balance resulting from excess calorie intake relative to energy expenditure [2]. A key regulator of energy expenditure is thermogenesis [3]. While white adipose tissue (WAT) primarily functions as an energy store, brown adipose tissue (BAT) dissipates energy through mitochondrial oxidation and thermogenesis mediated by uncoupling protein 1 (UCP1) [4, 5]. BAT is predominantly located in the interscapular region, but WAT also contains pockets of BAT-like adipocytes, known as beige cells. The process by which WAT undergoes a phenotypic switch to brown or beige adipocytes is called adipose tissue browning, which plays a crucial role in the pathogenesis of cancer cachexia and kidney failure [6–8]. Importantly, BAT activity is markedly reduced in individuals with obesity, suggesting that impaired thermogenic capacity may contribute to the development or maintenance of obesity [9]. Recent research shows that BAT activation enhances glucose and lipid clearance from circulation, thereby improving insulin resistance and β-cell function [10, 11]. Therefore, the induction of adipose tissue browning is considered a promising therapeutic strategy for obesity and obesity-related type 2 diabetes.

Classical approaches to induce adipose tissue browning, such as cold exposure and β-adrenergic agonist treatment, have shown potential but are substantially limited in clinical use due to the risk of adverse effects [12]. Thiazolidinediones, effective drugs for type 2 diabetes, have also been shown to induce adipose tissue browning by activating the nuclear receptor peroxisome proliferator-activated receptor γ [13]. However, the benefits of thiazolidinediones are often offset by side effects, including bone loss and congestive heart failure. In this context, parathyroid hormone (PTH) and tumor-derived PTH-related protein (PTHrP) have been identified as potent inducers of adipose tissue browning [7, 8, 14]. Notably, recombinant human PTH(1–34), known as teriparatide, is already used to treat osteoporosis [15], making it a promising therapeutic option to induce adipose tissue browning. This approach is particularly relevant for patients with type 2 diabetes, as they are at an increased risk of fractures [16]. In this study, we aimed to explore the therapeutic potential of PTH(1–34) as an inducer of adipose tissue browning in ob/ob mice, a leptin-deficient model of obesity and type 2 diabetes. Our findings reveal an unexpected role of PTH in the regulation of food intake and provide insights into how the effects of PTH on adipose tissue browning and systemic energy balance can be altered under different disease conditions.

## Methods

### Animal experiments

Five-week-old male ob/ob mice and their WT littermates (Oriental Yeast Co., Tokyo, Japan) were housed under standard laboratory conditions (23 ± 2 °C, 55 ± 10% humidity, 12-hour light/dark cycle), with free access to tap water and standard chow unless otherwise specified. After a 1-week acclimation period, 6-week-old mice were used for the experiments described below. All animal experiments were approved by the Institutional Animal Care and Use Committee of Tokai University School of Medicine.

To evaluate the acute effects of PTH, a single subcutaneous injection of either vehicle (0.9% saline containing 0.01 mM β-mercaptoethanol and 0.1 mM acetic acid) or human PTH(1–34) (Bachem) at doses of 80, 400, or 1000 µg/kg was administered to 6-week-old ob/ob mice and their WT littermates. Two hours after injection, adipose tissues were harvested for analysis of thermogenic gene expression. To assess the impact of PTH on glucose metabolism, serum samples were collected from ob/ob mice at 0, 2, 4, 8, and 24 h following a single injection of either vehicle or PTH (400–1000 µg/kg).

To investigate the long-term effects of PTH, 6-week-old ob/ob mice and their WT littermates received daily subcutaneous injections of either vehicle or PTH (400 µg/kg) for four weeks. Body weight and daily food intake were recorded weekly. At the end of the treatment period, after overnight fasting, serum and tissue samples were collected. Glucose tolerance and insulin tolerance tests were performed in a separate cohort.

To assess the effects of PTH on energy metabolism independent of food intake, an additional four-week pair-feeding experiment was conducted using ob/ob mice only. Each day, the PTH-treated group received the same amount of food consumed by the vehicle group on the previous day. All mice had free access to tap water.

### Biochemical analysis

Serum levels of glucose, cholesterol, and triglyceride were measured using enzymatic colorimetric methods (FUJIFILM Wako Chemicals, Osaka, Japan). Serum calcium and phosphorus levels were determined using standard laboratory methods. Serum insulin (Morinaga, Yokohama, Japan) and plasma levels of active and desacyl ghrelin (LSI Medience Corporation, Tokyo) were measured using commercially available ELISA kits according to the manufacturers’ instructions.

### Glucose and insulin tolerance tests

GTT and ITT were performed to assess glucose homeostasis. For the GTT, mice were fasted overnight and then injected intraperitoneally with glucose (2 g/kg body weight). For the ITT, mice were fasted for 4 h and injected intraperitoneally with insulin (2 U/kg body weight). Serum glucose levels were measured at 0, 15, 30, 45, 60, 90, and 120 min after injection in both tests.

### Grip strength

Forelimb grip strength was measured using the GPM-101B (Melquest, Toyama, Japan). Each mouse grasped a grip bar while its tail was pulled horizontally to the rear. The maximum grip strength was recorded just before the mouse released the grip.

### Histology

Adipose tissues (eWAT, iWAT, and iBAT), liver, and gastrocnemius muscle were fixed in 10% formalin, embedded in paraffin, sectioned, and stained with hematoxylin and eosin (H&E) using standard protocols. Liver sections were also stained with Oil Red O to assess lipid accumulation. Immunohistochemistry for UCP1 was performed in adipose tissues using an anti-UCP1 antibody (Abcam, ab10983) after antigen retrieval with 0.1% trypsin.

### µCT analysis

Excised tibiae were analyzed using the LaTheta LCT-200 scanner (Hitachi Aloka Medical, Tokyo, Japan). BMD and bone strength index were quantified with the LaTheta software.

### qPCR

Total RNA was extracted from adipose tissues, liver, gastrocnemius muscle, stomach, hypothalamus, calvaria, and kidney using TRIzol reagent (Thermo Fisher Scientific, Waltham, MA). For calvaria samples, tissues were homogenized in TRIzol reagent with 5-mm stainless steel beads using a Shake Master NEO (Bio Medical Sciences, Tokyo, Japan). Complementary DNA (cDNA) was synthesized from 0.5 µg of RNA using the SuperScript IV VILO Master Mix with ezDNase Enzyme (Thermo Fisher Scientific). qPCR was performed on a StepOnePlus System (Applied Biosystems) using the TaqMan One-Step RT-PCR Master Mix Reagents kit (Thermo Fisher Scientific). *Glyceraldehyde-3-phosphate dehydrogenase (GAPDH)* was used as an internal control.

### Statistical analysis

Group differences were assessed by Student’s t-test or one-way ANOVA with Dunnett’s post hoc test or Tukey’s post hoc test, as appropriate. Longitudinal data were analyzed using mixed-effects models. *P* < 0.05 was considered statistically significant. All analyses were performed using GraphPad Prism 8 and IBM SPSS Statistics 24.

## Results

### Expression of PTH receptor in adipose tissues and other organs

We first examined the tissue-specific expression of *parathyroid hormone 1 receptor (Pth1r)*, which encodes the receptor for PTH, in ob/ob mice and their wild-type (WT) littermates (Supplemental Fig. 1). As expected, *Pth1r* was highly expressed in bone and kidney. Expression was also detected in epididymal WAT (eWAT; a visceral fat depot), inguinal WAT (iWAT; a form of subcutaneous fat), and interscapular BAT (iBAT), with a trend toward reduced expression in eWAT and iWAT of ob/ob mice. Notably, *Pth1r* expression was also observed in the stomach.

### PTH drives thermogenic gene expression in adipose tissues of ob/ob mice

**Fig. 1:**
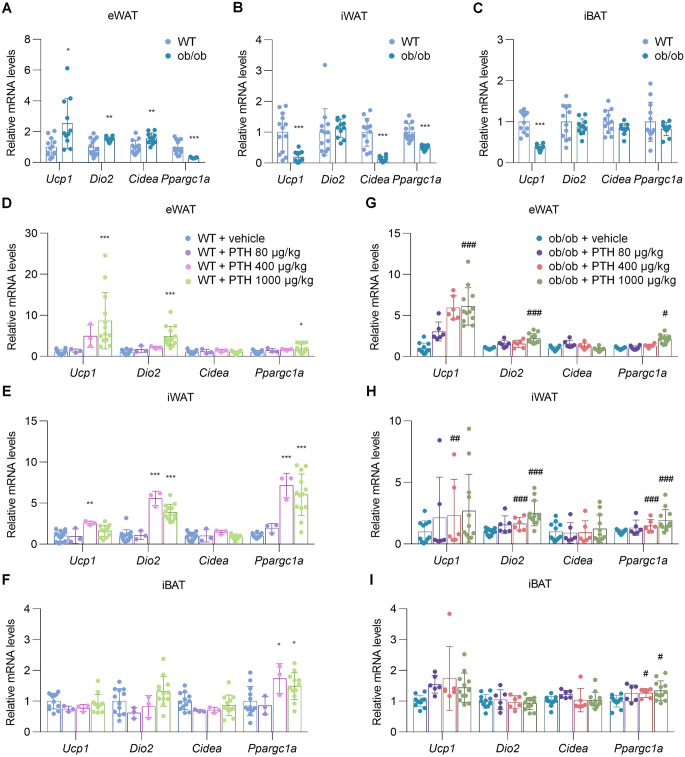
Acute injection of PTH promotes thermogenic gene expression in adipose tissues of ob/ob mice. ob/ob mice and their WT littermates were administered a single subcutaneous injection of vehicle or PTH(1–34) at the indicated doses. Two hours after injection, mRNA levels of thermogenic genes in adipose tissue were measured by qPCR. (**A–C**) Thermogenic gene expression in eWAT (**A**), iWAT (**B**), and iBAT (**C**) was compared between vehicle-treated ob/ob mice and WT littermates (WT, *n* = 14; ob/ob, *n* = 12). (**D–I**) Thermogenic gene expression in eWAT (**D**,** G**), iWAT (**E**,** H**), and iBAT (**F**,** I**) was examined in WT mice (**D–F**) and ob/ob mice (**G–I**) following injection of vehicle or PTH. WT: vehicle (*n* = 13), PTH 80 µg/kg (*n* = 3), 400 µg/kg (*n* = 3), 1000 µg/kg (*n* = 13); ob/ob: vehicle (*n* = 11), PTH 80 µg/kg (*n* = 6), 400 µg/kg (*n* = 6), 1000 µg/kg (*n* = 11). PTH 80 and 400 µg/kg groups were additionally included to evaluate dose-dependent responses. Data are presented as mean ± SD. ^*^P < 0.05, ^**^P < 0.01, ^***^P < 0.001 vs. vehicle-treated WT littermates; ^#^*P* < 0.05, ^##^*P* < 0.01, ^###^*P* < 0.001 vs. vehicle-treated ob/ob mice. Statistical analysis was performed using one-way ANOVA with Dunnett’s post hoc test

To examine the acute effects of PTH on thermogenic gene expression in adipose tissues, a single subcutaneous injection of either vehicle or PTH(1–34) was administered to ob/ob mice and their WT littermates. Two hours after injection, the expression of thermogenic genes was assessed in adipose tissues. We first compared thermogenic gene expression between ob/ob mice and WT littermates treated with vehicle (Fig. 1, A–C). Consistent with previous reports [17] and the known thermogenic impairment of this mouse model [18], *Ucp1* expression in iBAT was significantly reduced in ob/ob mice compared to WT littermates (Fig. 1C). A similar reduction in thermogenic gene expression was observed in iWAT of ob/ob mice (Fig. 1B), whereas eWAT exhibited a contrasting trend toward increased expression (Fig. 1A). In WT littermates, PTH significantly upregulated *Ucp1*, *type II iodothyronine deiodinase (Dio2)*, and *peroxisome proliferator-activated receptor gamma coactivator 1-alpha (Ppargc1a)* in both eWAT and iWAT in a dose-dependent manner (Fig. 1, D and E). A similar induction of these genes was observed in ob/ob mice, although the effects tended to be less pronounced compared to WT littermates (Fig. 1, G and H). PTH also modestly increased *Pgc1a* expression in iBAT of both ob/ob mice and WT littermates (Fig. 1, F and I). Serum glucose levels were unaffected by acute PTH administration in ob/ob mice (Supplemental Fig. 2).

### Sustained PTH treatment leads to unexpected weight gain without inducing adipose tissue browning in ob/ob mice

**Fig. 2:**
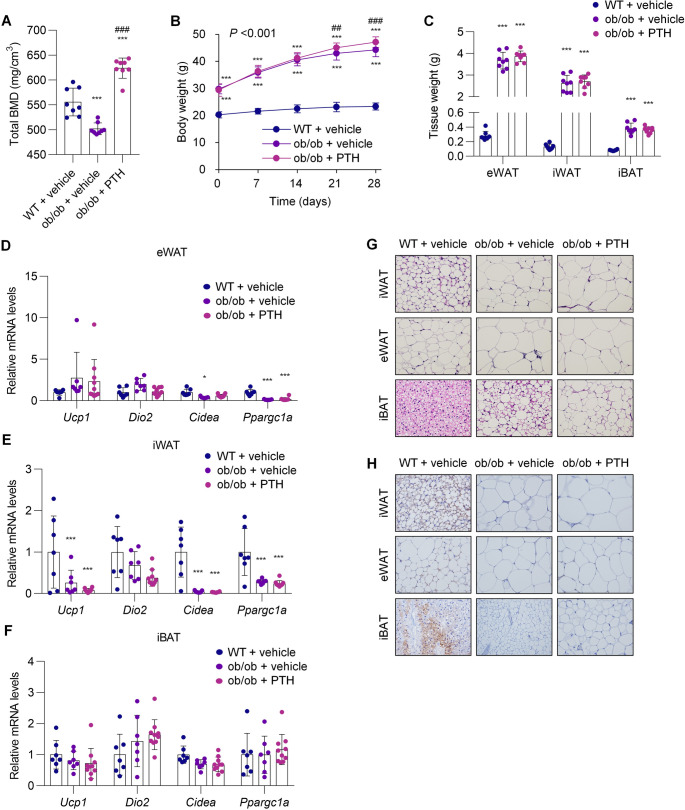
Sustained PTH treatment leads to unexpected weight gain without inducing adipose tissue browning in ob/ob mice. ob/ob mice were treated with either vehicle or PTH(1–34) (400 µg/kg) by daily injection for four weeks; WT littermates received vehicle only. (**A**) Total tibial BMD was assessed by µCT at the end of treatment. (**B**) Body weight was monitored throughout the treatment period. (**C**) Weights of individual adipose depots were measured at the end of treatment. (**D–F**) Expression of thermogenic genes in eWAT (**D**), iWAT (**E**), and iBAT (**F**) was assessed by qPCR. (**A–C**) *n* = 8 per group; (**D–F**) *n* = 7–10 per group. Data are presented as mean ± SD. ^*^P < 0.05, ^***^P < 0.001 vs. vehicle-treated WT littermates; ^##^*P* < 0.01, ^###^*P* < 0.001 vs. vehicle-treated ob/ob mice. The numerical *P* value shown is for the interaction between time and group. Statistical analysis was performed using one-way ANOVA with Tukey’s post hoc test (**A**,** C–F**) or mixed-effects models (**B**). (**G**) H&E staining and (**H**) UCP1 immunohistochemistry were performed on sections from individual adipose depots collected at the end of treatment

To determine whether PTH-induced thermogenic gene expression contributes to the improvement of obesity and metabolic homeostasis, we administered either vehicle or PTH to ob/ob mice for four weeks. As a first step, we confirmed the biological efficacy of PTH by assessing its known anabolic effects on bone formation [19]. µCT analysis of the tibia revealed a significant reduction in total bone mineral density (BMD) in ob/ob mice compared to their WT littermates (Fig. 2A). PTH treatment significantly increased total BMD, with a preferential effect on trabecular rather than cortical bone (Supplemental Fig. 3, A–C), and was accompanied by improved biomechanical properties (Supplemental Fig. 3D). Serum calcium and phosphorus levels were not altered by PTH treatment (Supplemental Fig. 3, E and F).

We then focused on the metabolic consequences of sustained PTH administration. To our surprise, PTH treatment further increased the already elevated body weight of ob/ob mice compared with vehicle-treated counterparts (Fig. 2B), although no detectable changes were observed in the weights of iWAT, eWAT, or iBAT (Fig. 2C). Histological analysis revealed large unilocular lipid droplets in adipocytes across all depots in ob/ob mice relative to WT littermates (Fig. 2G). PTH did not reduce droplet size in iWAT or eWAT and instead promoted an increase in droplet size in iBAT. Thermogenic gene expression in adipose tissues of ob/ob mice was not induced by PTH treatment (Fig. 2D–F), and immunohistochemistry further confirmed the absence of UCP1 induction (Fig. 2H). Although previous studies have suggested that PTH may contribute to adipocyte-mediated muscle wasting [7, 8], we did not observe the induction of muscle atrophy-related genes, muscle weakness, or histological evidence of muscle fiber atrophy following PTH treatment in this model (Supplemental Fig. 4, A–C).

### PTH treatment does not improve glucose and lipid metabolism and exacerbates hyperphagia in ob/ob mice

**Fig. 3:**
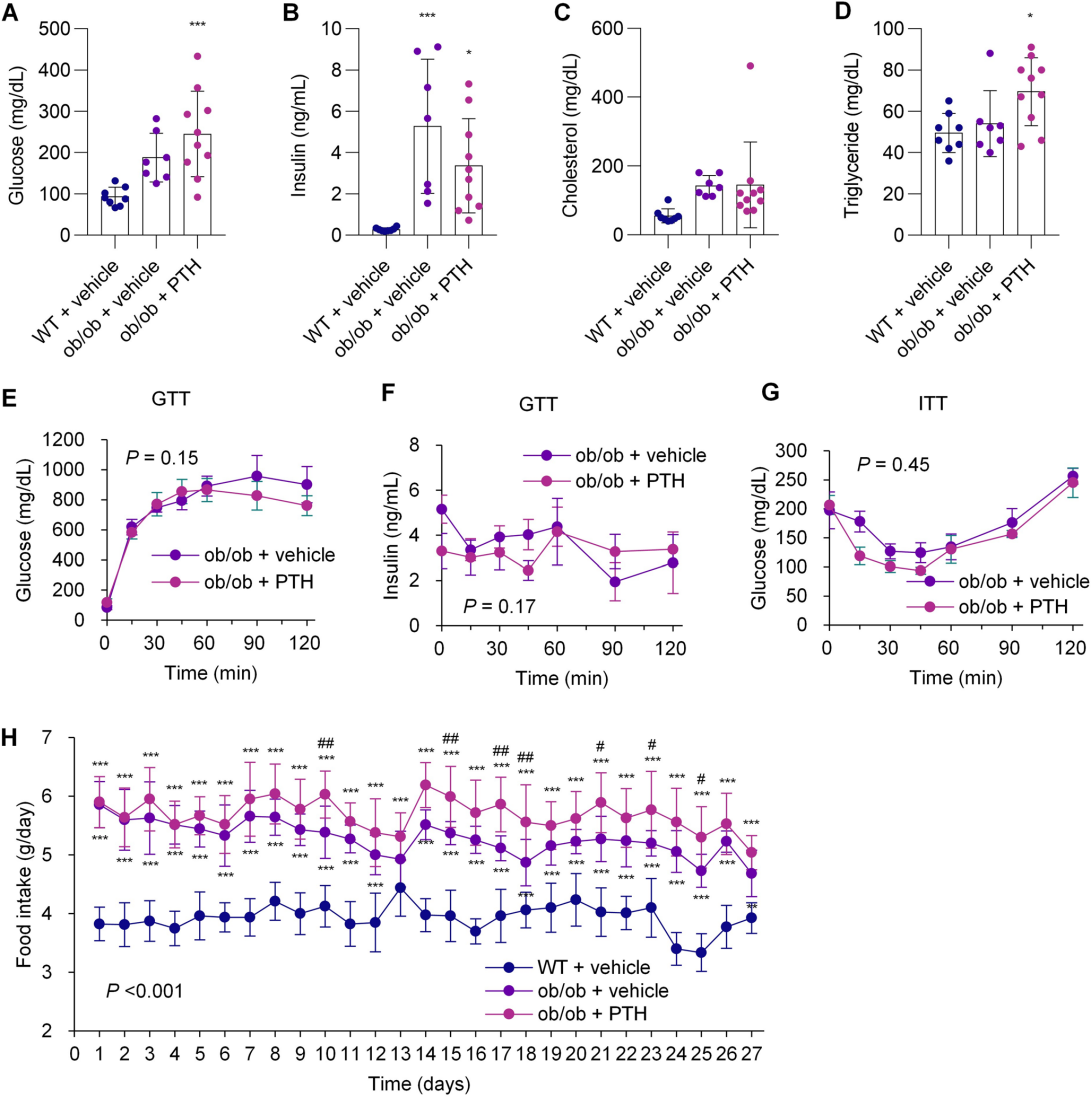
PTH treatment does not improve glucose and lipid metabolism and exacerbates hyperphagia in ob/ob mice. ob/ob mice were treated with either vehicle or PTH(1–34) (400 µg/kg) by daily injection for four weeks; WT littermates received vehicle only. (**A-D**) Fasting serum glucose (**A**), insulin (**B**), cholesterol (**C**) and triglycerides (**D**) were measured at the end of treatment. (**E–G**) GTT measuring glucose (**E**) and insulin (**F**) and ITT (**G**) were performed after four weeks of treatment. (**H**) Food intake was monitored throughout the treatment period. (**A–D**,** H**) *n* = 7–10 per group; (**E–G**) *n* = 6 per group. Data are presented as mean ± SD (**A–D**,** H**) or mean ± SE (**E–G**). ^*^P < 0.05, ^***^P < 0.001 vs. vehicle-treated WT littermates; ^#^*P* < 0.05, ^##^*P* < 0.01 vs. vehicle-treated ob/ob mice. The numerical *P* values shown are for the interaction between time and group. Statistical analysis was performed using one-way ANOVA with Tukey’s post hoc test (**A–D**) or mixed-effects models (**E–H**)

We further evaluated the effects of PTH on glucose and lipid metabolism. Contrary to our initial expectations, serum glucose tended to be higher in PTH-treated ob/ob mice than in vehicle-treated ob/ob mice in both fasting and random-fed states (Fig. 3A and Supplemental Fig. 5A). Insulin levels were comparable between the two groups in both states (Fig. 3B and Supplemental Fig. 5B). Cholesterol levels showed a tendency to be higher in PTH-treated ob/ob mice in the random-fed condition (Fig. 3C and Supplemental Fig. 5C), while triglyceride levels were increased in both fasting and random-fed states (Fig. 3D and Supplemental Fig. 5D). To further assess insulin secretory responses and insulin sensitivity, we performed glucose tolerance tests (GTT) and insulin tolerance tests (ITT), which revealed no differences in these responses (Fig. 3, E–G). PTH also had no apparent effect on hepatic expression of *Tnf*, which encodes tumor necrosis factor-alpha, or the histological features of hepatic steatosis (Supplemental Fig. 6, A and B).

To investigate the cause of weight gain in PTH-treated ob/ob mice in the absence of adipose tissue browning, we analyzed food intake over time. We found that ob/ob mice were already hyperphagic compared to their littermates, and PTH treatment showed a trend toward further increasing food intake in ob/ob mice (Fig. 3G).

### PTH does not improve obesity or glucose metabolism in ob/ob mice even under pair-feeding conditions

**Fig. 4:**
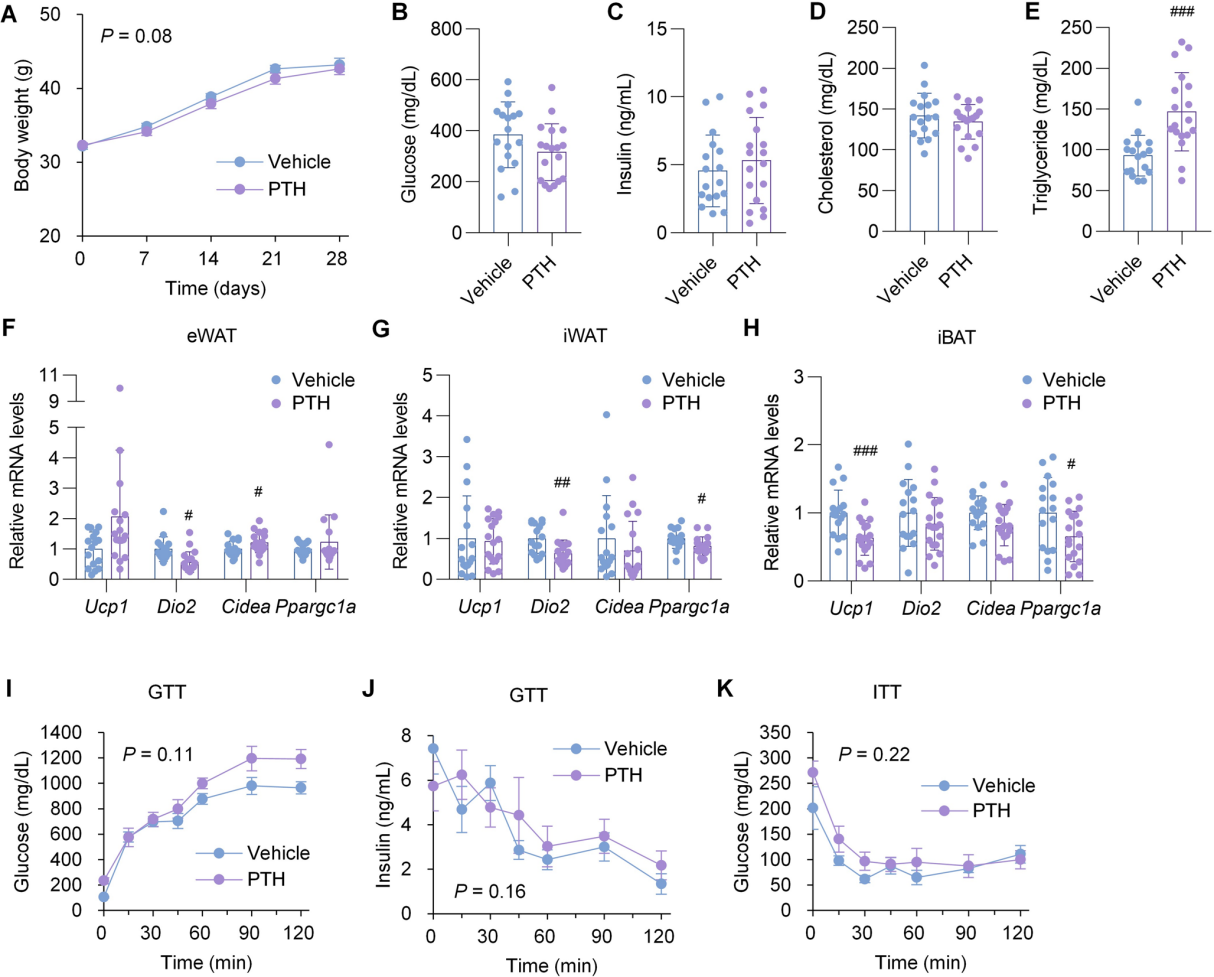
PTH does not improve obesity or glucose metabolism in ob/ob mice even under pair-feeding conditions. ob/ob mice were treated with either vehicle or PTH(1–34) (400 µg/kg) by daily injection for four weeks, with PTH-treated mice pair-fed to vehicle-treated controls. (**A**) Body weight was monitored throughout the treatment period. (**B–E**) Fasting serum glucose (**B**), insulin (**C**), cholesterol (**D**), and triglycerides (**E**) were measured at the end of treatment. (**F–H**) Expression of thermogenic genes in eWAT (**F**), iWAT (**G**), and iBAT (**H**) was assessed by qPCR. (**I–K**) GTT measuring glucose (**I**) and insulin (**J**) and ITT (**K**) were performed after four weeks of treatment. (**A–H**) *n* = 16–20 per group; (**I–K**) *n* = 6 per group. Data are presented as mean ± SD (**A–H**) or mean ± SE (**I–K**). ^#^*P* < 0.05, ^##^*P* < 0.01, ^###^*P* < 0.001 vs. vehicle-treated ob/ob mice. The numerical *P* values shown are for the interaction between time and group. Statistical analysis was performed using mixed-effects models (**A**, **I–K**) or Student’s t-test (**B–H**)

Based on our findings that PTH administration in ob/ob mice induced hyperphagia without improving obesity, glucose metabolism, or adipose tissue browning, we conducted an additional four-week pair-feeding experiment in which ob/ob mice received either PTH or vehicle. Under these conditions, PTH-treated ob/ob mice showed only a slight trend toward reduced body weight compared to vehicle-treated counterparts (Fig. 4A), with no changes in fasting serum glucose or insulin levels (Fig. 4, B and C). PTH treatment appeared to slightly increase triglyceride levels, while cholesterol levels remained unchanged (Fig. 4, D and E). Furthermore, PTH treatment failed to induce thermogenic gene expression in adipose tissue (Fig. 4, F–H) and had no effect on insulin secretory responses or insulin sensitivity, as assessed by GTT and ITT (Fig. 4, I–K).

### Ghrelin signaling may contribute to PTH-induced hyperphagia in ob/ob mice

**Fig. 5:**
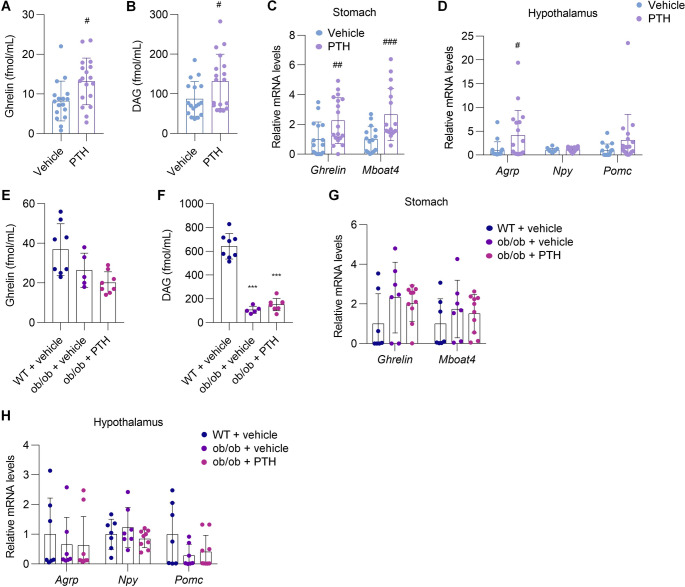
Ghrelin signaling may contribute to PTH-induced hyperphagia in ob/ob mice. (**A–D**) Appetite-regulating hormone profiles were assessed in pair-fed ob/ob mice treated with vehicle or PTH(1–34) for four weeks. Plasma levels of active ghrelin (**A**) and des-acyl ghrelin (DAG) (**B**) were measured at the end of treatment. Gastric expression of ghrelin-related genes (**C**) and hypothalamic expression of orexigenic and anorexigenic neuropeptide genes (**D**) were analyzed by qPCR. (**E–H**) Appetite-regulating hormone profiles were assessed in ad libitum ob/ob mice treated with vehicle or PTH(1–34) for four weeks. Plasma levels of active ghrelin (**E**) and DAG (**F**) were measured at the end of treatment. Gastric expression of ghrelin-related genes (**G**) and hypothalamic expression of orexigenic and anorexigenic neuropeptide genes (**H**) were analyzed by qPCR. (**A–D**) *n* = 16–18 per group; (**E–H**) *n* = 5–10 per group. Data are presented as mean ± SD. ^***^P < 0.001 vs. vehicle-treated WT littermates; ^#^*P* < 0.05, ^##^*P* < 0.01, ^###^*P* < 0.001 vs. vehicle-treated ob/ob mice. Statistical analysis was performed using Student’s t-test (**A–D**) or one-way ANOVA with Tukey’s post hoc test (**E–H**)

Lastly, we sought to elucidate the mechanism underlying PTH-induced food intake in ob/ob mice. Ghrelin, a hormone secreted by the stomach, is well known for its orexigenic properties [18]. In pair-fed ob/ob mice, plasma levels of both active ghrelin and des-acyl ghrelin (DAG) were significantly elevated in PTH-treated mice compared to controls (Fig. 5, A and B). This was accompanied by upregulation of gastric *Ghrelin* and *Mboat4*, which encodes ghrelin-O-acyltransferase (GOAT), the enzyme required for ghrelin activation (Fig. 5C). Furthermore, hypothalamic expression of *agouti-related peptide (Agrp)*—a neuropeptide induced by ghrelin that promotes food intake [20]—was significantly increased in PTH-treated ob/ob mice (Fig. 5D). Notably, these changes were not observed in ob/ob mice fed ad libitum during PTH treatment (Fig. 5, E–H), likely due to feedback suppression of ghrelin signaling resulting from increased food intake. Taken together, these findings suggest that enhanced gastric ghrelin production mediates PTH-induced hyperphagia and weight gain in ob/ob mice, which may, in turn, attenuate the thermogenic actions of PTH on adipose tissue.

## Discussion

Obesity is a major global health concern that contributes to diabetes and other metabolic disorders, primarily driven by an imbalance between caloric intake and energy expenditure [1, 2]. This study investigated the effects of PTH, which has been shown to promote thermogenesis and induce browning of adipose tissue [7, 8, 14], in ob/ob mice—a well-established model of obesity-associated insulin resistance and type 2 diabetes. While acute PTH administration transiently upregulated thermogenic gene expression in adipose tissue, chronic treatment unexpectedly exacerbated obesity and failed to improve adiposity or glucose metabolism.

The unexpected finding that chronic PTH administration failed to induce adipose tissue browning and instead exacerbated weight gain in ob/ob mice appears to be largely attributable to enhanced food intake. This suggests that hyperphagia, a hallmark of ob/ob mice resulting from leptin deficiency, was further exacerbated by PTH treatment. However, even when caloric intake was controlled through pair-feeding experiments, PTH conferred minimal effects on body weight and glucose metabolism. In these experiments, ob/ob mice treated with PTH remained hyperphagic and exhibited obesity to a similar extent as the vehicle-treated group. The resulting adiposity may have blunted the browning-inducing effects of PTH on adipose tissue through certain mechanisms. Although the precise molecular pathways remain unclear, we confirmed reduced expression of *Pth1r* in eWAT and iWAT of ob/ob mice, which may impair PTH signaling in adipocytes and contribute to the minimal effects of PTH on adipose tissue browning. In this context, perhaps an even more important point to recognize is that leptin is involved not only in appetite suppression but also in promoting thermogenesis in adipocytes [21, 22]. Adipose tissue in ob/ob mice is known to exhibit markedly reduced thermogenic activity [18], as evidenced by our findings of significantly reduced thermogenic gene expression in both iBAT and iWAT. It is possible to hypothesize that this unique feature of ob/ob adipocytes may be involved in the blunted response to PTH-induced adipose tissue browning.

To elucidate the mechanism underlying the further increase in food intake in ob/ob mice following PTH treatment, we examined appetite-regulating pathways. In pair-feeding experiments, PTH administration increased *ghrelin* expression in the stomach and elevated circulating ghrelin levels. This was paralleled by enhanced *Agrp* expression in the hypothalamus, a downstream response to ghrelin known to stimulate appetite [20]. These findings underscore a novel neuroendocrine axis through which PTH amplifies peripheral-to-central appetite-promoting signals, resulting in increased caloric intake. However, these changes were not observed in ob/ob mice fed ad libitum during PTH treatment. This discrepancy may be attributable to feedback suppression of ghrelin signaling secondary to increased food intake, although this remains speculative and requires further validation. Furthermore, although our findings suggest that PTH may increase gastric ghrelin production, the underlying mechanism remains unclear. Given the expression of *Pth1r* in the stomach, it is conceivable that PTH may act directly on the stomach to stimulate ghrelin production. However, this hypothesis remains unproven and warrants further investigation.

The lack of metabolic benefits and the unexpected weight gain observed with PTH treatment in ob/ob mice stand in sharp contrast to prior findings from animal models of catabolic disease, such as kidney failure and cancer cachexia [7, 8, 14], as well as from clinical studies in these populations [23–25]. Several factors may underlie this discrepancy. One possibility is the distinct pattern of circulating PTH levels. While circulating levels of PTHrP in cancer and PTH in kidney failure are chronically elevated, repeated subcutaneous administration of PTH in the present study is expected to produce transient peaks followed by a rapid decline. These kinetic differences in PTH exposure may differentially influence the induction of adipose tissue browning and its downstream metabolic effects. Another plausible explanation relates to differences in the underlying pathophysiology. In kidney failure or cancer, reduced appetite and associated malnutrition are major clinical concerns, and under such conditions, increased energy expenditure driven by PTH/PTHrP or other catabolic stimuli may impose a significant metabolic burden. In contrast, in the context of obesity, which is characterized by hyperphagia and excessive caloric intake, the relative impact of PTH on systemic energy balance may be substantially limited, particularly given the diminished effect of PTH on thermogenic gene expression, as observed in the present study. Thus, the differential effects of PTH/PTHrP across distinct pathological contexts underscore the complex and context-dependent nature of its metabolic actions.

One important limitation of this study is that we exclusively used ob/ob mice as a model of obesity. Unlike common forms of obesity characterized by leptin resistance [26], ob/ob mice exhibit congenital leptin deficiency. Therefore, the reduced responsiveness to PTH, as well as its effects on ghrelin and feeding behavior, may be specific to this model and not fully generalizable to other forms of obesity, such as diet-induced obesity models. Further studies using alternative obesity models will be important to clarify the generalizability of our findings.

In summary, chronic PTH administration in ob/ob mice failed to induce adipose browning or improve metabolic parameters, instead exacerbating hyperphagia. These findings highlight the limited therapeutic potential of PTH in obesity and raise the intriguing possibility that PTH not only regulates energy expenditure but also influences caloric intake via central appetite circuits. Our data underscore the importance of considering the disease context when evaluating the metabolic effects of PTH and suggest that the actions of PTH or PTHrP to enhance energy expenditure may differ significantly depending on the underlying nutritional and hormonal environment.

## Supplementary Information

Below is the link to the electronic supplementary material.

Supplementary Material 1 (PDF 489 KB)

## Data Availability

The datasets generated and/or analyzed during the current study are available from the corresponding author on reasonable request.
