# Supplementary material for: Parathyroid hormone enhances appetite and fails to reduce adiposity in ob/ob mice
Source: Pflugers Arch. 2026 Jun 3;478(6):52. doi: 10.1007/s00424-026-03183-y (PMC13230290; doi:10.1007/s00424-026-03183-y)
Supplement: Supplementary file 1 — Supplementary Material 1 (PDF 489 KB) [file 424_2026_3183_MOESM1_ESM.pdf]

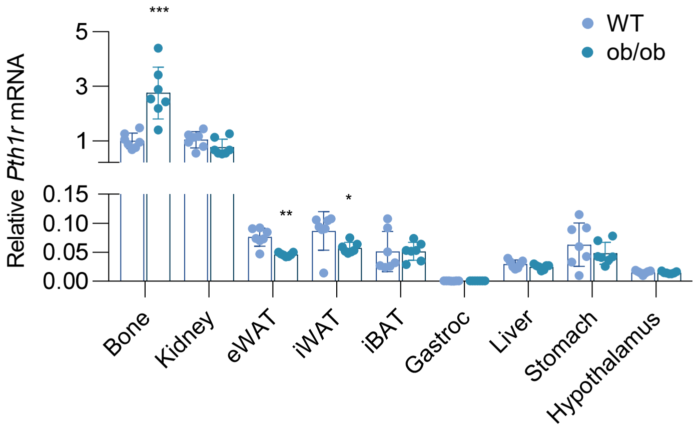

Supplemental Figure 1. Expression of PTH receptor in adipose tissues and other organs. mRNA levels of *Pth1r* were quantified by qPCR in various organs from ob/ob mice and their WT littermates. n = 7 per group. Data are presented as mean ± SD. $^{*}P$ <0.05, $^{**}P$ <0.01, $^{***}P$ <0.001 vs. WT littermates. Statistical analysis was performed using Student's t-test.

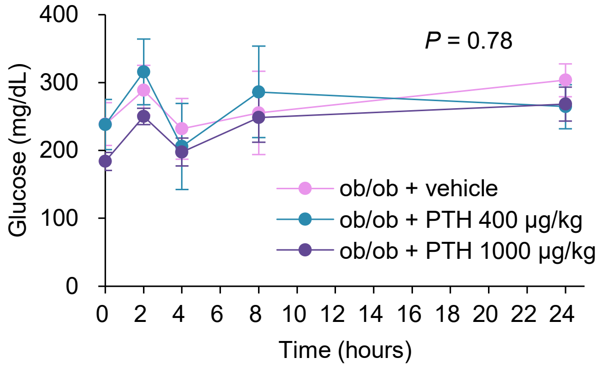

Supplemental Figure 2. Acute injection of PTH does not affect serum glucose levels in ob/ob mice. Serum glucose levels were monitored over time in ob/ob mice following injection of vehicle or PTH. n = 6 per group. Data are presented as mean ± SD. *P* value is for the interaction between time and group. Statistical analysis was performed using mixed-effects models.

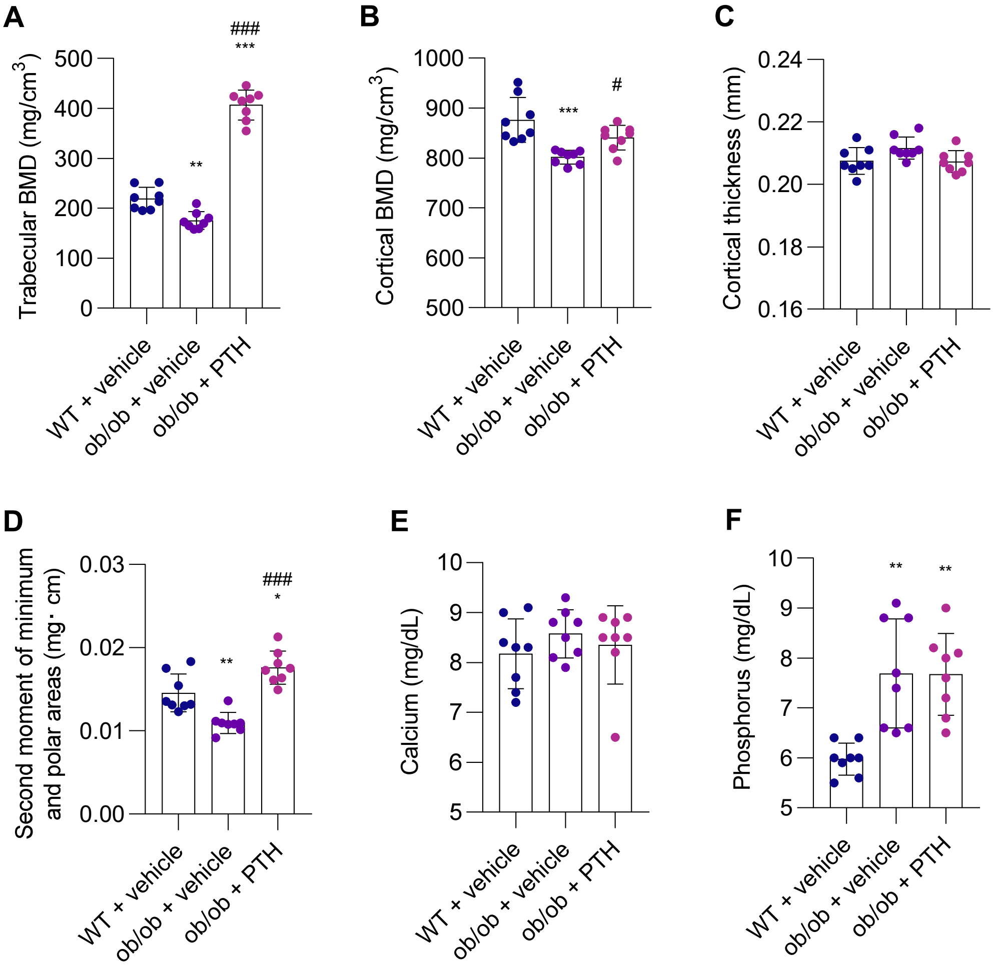

Supplemental Figure 3. Sustained PTH treatment improves trabecular BMD and mechanical strength of the tibia in ob/ob mice. ob/ob mice were treated with either vehicle or PTH(1–34) (400 μg/kg) by daily injection for four weeks; WT littermates received vehicle only. (**A–C**) Trabecular BMD (**A**), cortical BMD (**B**), and cortical thickness (**C**) were assessed by μCT at the end of treatment. (**D**) Bone strength index (second moment of minimum and polar areas) was calculated from μCT data using analysis software. (**E** and **F**) Serum calcium (**E**) and phosphorus (**F**) were measured at the end of treatment. n = 8 per group. Data are presented as mean ± SD. $^{*}P$ <0.05, $^{***}P$ <0.001 vs. vehicle-treated WT littermates; $^{\#}P$ <0.05, $^{\#\#\#}P$ <0.001 vs. vehicle-treated ob/ob mice. Statistical analysis was performed using one-way ANOVA with Tukey's post hoc test.

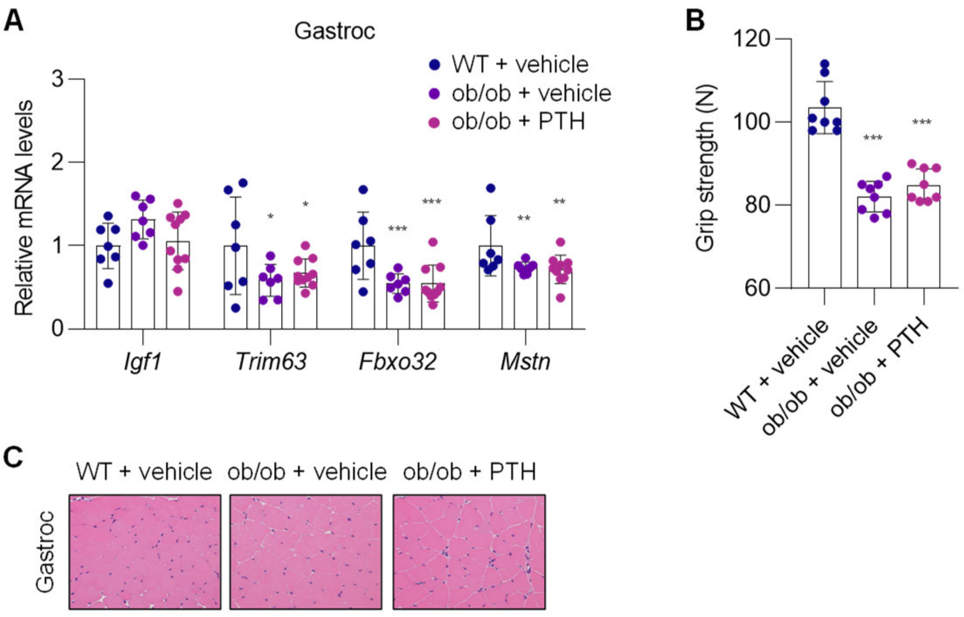

Supplemental Figure 4. PTH treatment does not affect muscle gene expression or muscle strength in ob/ob mice. ob/ob mice were treated with either vehicle or PTH(1–34) (400 µg/kg) by daily injection for four weeks; WT littermates received vehicle only. (**A**) Expression of muscle atrophy-related genes in the gastrocnemius muscle was assessed by qPCR after four weeks of treatment. (**B**) Grip strength was assessed at the end of treatment. n = 7–10 per group. Data are presented as mean ± SD. $^{*}P$ <0.05, $^{**}P$ <0.01, $^{***}P$ <0.001 vs. vehicle-treated WT littermates. Statistical analysis was performed using one-way ANOVA with Tukey's post hoc test. (**C**) H&E staining was performed on sections of gastrocnemius muscle collected at the end of treatment.

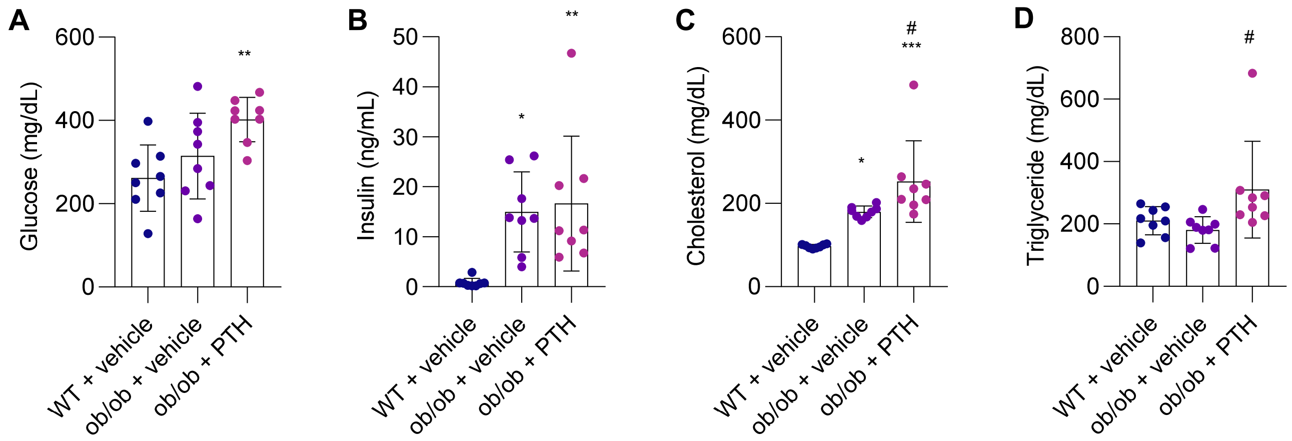

Supplemental Figure 5. PTH treatment does not improve glucose and lipid metabolism in ob/ob mice. ob/ob mice were treated with either vehicle or PTH(1–34) (400 µg/kg) by daily injection for four weeks; WT littermates received vehicle only. (**A-D**) Random serum glucose (**A**), insulin (**B**), cholesterol (**C**) and triglycerides (**D**) were measured at the end of treatment. n = 8 per group. Data are presented as mean ± SD. $^{*}P$ <0.05, $^{**}P$ <0.01, $^{***}P$ <0.001 vs. vehicle-treated WT littermates; $^{\#}P$ <0.05 vs. vehicle-treated ob/ob mice. Statistical analysis was performed using one-way ANOVA with Tukey's post hoc test.

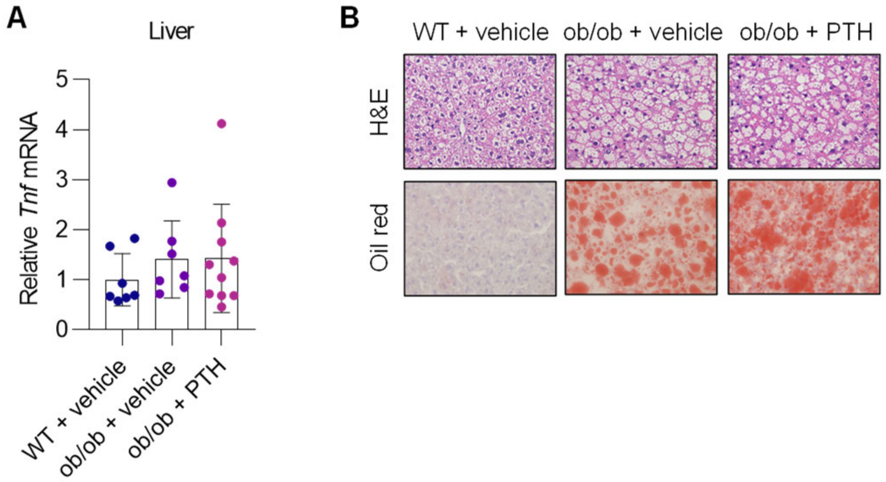

Supplemental Figure 6. PTH treatment does not affect hepatic steatosis in ob/ob mice. ob/ob mice were treated with either vehicle or PTH(1–34) (400 µg/kg) by daily injection for four weeks; WT littermates received vehicle only. (**A**) Hepatic *Tnf* mRNA levels were assessed by qPCR after four weeks of treatment. n = 7–10 per group. Data are presented as mean ± SD. Statistical analysis was performed using one-way ANOVA with Tukey's post hoc test. (**B**) H&E and Oil Red O staining were performed on sections of liver collected at the end of treatment.
